# Transcriptional inhibition of miR-486-3p by BCL6 upregulates Snail and induces epithelial–mesenchymal transition during radiation-induced pulmonary fibrosis

**DOI:** 10.1186/s12931-022-02024-7

**Published:** 2022-04-28

**Authors:** Ziyan Yan, Xingkun Ao, Xinxin Liang, Zhongmin Chen, Yuhao Liu, Ping Wang, Duo Wang, Zheng Liu, Xiaochang Liu, Jiaojiao Zhu, Shenghui Zhou, Pingkun Zhou, Yongqing Gu

**Affiliations:** 1 Beijing Key Laboratory for Radiobiology, Beijing Institute of Radiation Medicine, Beijing, China; 2 Hengyang Medical College, University of South China, Hengyang, Hunan China; 3 PLA Rocket Force Characteristic Medical Center, Beijing, China; 4 School of Public Health, University of South China, Hengyang, Hunan China

**Keywords:** miR-485-3p, EMT, Snail, BCL6, Radiation-induced pulmonary fibrosis

## Abstract

**Background:**

Ionizing radiation (IR) can induce pulmonary fibrosis by causing epithelial mesenchymal transition (EMT), but the exact mechanism has not been elucidated. To investigate the molecular mechanism of how radiation induces pulmonary fibrosis by altering miR-486-3p content and thus inducing EMT.

**Methods:**

The changes of miR-486-3p in cells after irradiation were detected by RT-qPCR. Western blot was used to detect the changes of cellular epithelial marker protein E-cadherin, mesenchymal marker N-cadherin, Vimentin and other proteins. The target gene of miR-486-3p was predicted by bioinformatics method and the binding site was verified by dual luciferase reporter system. In vivo experiments, adeno-associated virus (AAV) was used to carry miR-486-3p mimic to lung. Radiation-induced pulmonary fibrosis (RIPF) model was constructed by 25Gy^60^Co γ-rays. The structural changes of mouse lung were observed by HE and Masson staining. The expression of relevant proteins in mice was detected by immunohistochemistry.

**Results:**

IR could decrease the miR-486-3p levels in vitro and in vivo, and that effect was closely correlated to the occurrence of RIPF. The expression of Snail, which induces EMT, was shown to be restrained by miR-486-3p. Therefore, knockdown of Snail blocked the EMT process induced by radiation or knockdown of miR-486-3p. In addition, the molecular mechanism underlying the IR-induced miRNA level reduction was explored. The increased in BCL6 could inhibit the formation of pri-miR-486-3p, thereby reducing the levels of miR-486-3p in the alveolar epithelial cells, which would otherwise promote EMT and contribute to RIPF by targeting Snail.

**Conclusion:**

IR can exacerbate RIPF in mice by activating the transcription factor BCL6, which inhibits the transcription of miR-486-3p and decreases its content, which in turn increases the content of the target gene slug and triggers EMT.

## Background

The incidence of chest tumors, such as breast cancer and lung cancer, is increasing every year [1, 2]. Radiotherapy is frequently used for the clinical treatment of chest tumors, although certain side effects are inevitable. Radiation-induced pulmonary fibrosis (RIPF) is a familiar complication of radiotherapy conducted for chest tumors, which presents a high incidence, complex etiology, and an extremely poor prognosis [3–7]. It has been showed that the epithelial-mesenchymal transition (EMT) produces more than 30% of fibroblasts in pulmonary fibrosis models [8]. EMT refers to a process in which the epithelial cells acquire the morphology and features of mesenchymal cells. The decrease of E-cadherin and the increase of N-cadherin and Vimentin are used to indicate the occurrence of EMT. Despite the significance of the EMT process in RIPF, studies on this topic are scarce.

MicroRNAs (miRNAs) are believed to bind to the target messenger RNA’s 3′-untranslated region (3′-UTR) to regulate their expression [9]. MiRNAs are quite abundant in organisms, suggesting that they are critical to the development of disease [10–12]. However, the role of miRNAs specifically in RIPF remains to be elucidated so far. In our previous research, miR-486-3p was downregulated in RIPF mouse model through high-throughput sequencing [13]. miR-486-3p was confirmed to be expressed higher in patients with liver cirrhosis and mice with liver fibrosis. As a risk factor for liver fibrosis/cirrhosis of the liver, it mediated detoxification activity by lowering UGT1A [14]. PMN-MDSCs promote the NF-kappa B2 signal pathway by inhibiting miR-486-3p and accelerating the thyroid cancer cell invasion [15]. However, the precise involvement of miR-486-3p in RIPF has yet to be determined, and the underlying mechanism is still unknown.

Snail is known as the “master regulator” that globally regulates the EMT process [16, 17]. During cervical cancer metastasis, Snail formed complexes with PRMT5 and NuRD (MTA1) to promote EMT both in vivo and in vitro [18]. On colorectal cancer (CRC), THZ1 increases the EMT of CRC cells by enhancing the protein stability of Snail [19]. Furthermore, research has demonstrated that NF-κB can increase the stability of Snail to promote EMT and facilitate cell migration and invasion driven by inflammation [20]. Besides, Snail promotes EMT by increasing the expression level of tight junction protein Claudin-11, causing collective migration of squamous cell carcinoma [21]. Numerous experiments have established that Snail is a crucial factor in EMT and deserves further research.

In this study, we detected changes in miR-486-3p after irradiation. To probe the mechanism in this change, we analyzed the promoter region of miR-486-3p and proved a transcription factor, B cell lymphoma 6 protein (BCL6), to reduce miR-486-3p expression after IR. BCL6 is a member of the BTB/POZ zinc finger protein family [22–25] and is reported as a class of transcriptional inhibitors that can bind to specific sequences of target genes and inhibit the transcription of target genes [26–30]. It has also been reported to be highly correlated with invasiveness [31–35].

Our findings revealed that irradiation could activate BCL6, inhibit the transcription of miR-486-3p, increase the content of the target gene Snail, promotes EMT, and leads to RIPF. This study on irradiation led to the EMT, and RIPF provides a new way.

## Materials and methods

### Cell culture

A549 and BEAS-2B cells were purchased from the National Collection of Authenticated Cell Cultures and stored in our laboratory. Genetic information for all cell lines could find in the Cellosaurus database (https://web.expasy.org/cellosaurus/ accessed on 18 October 2021). All the cells were grown in high-glucose Dulbecco’s modified Eagle medium (DMEM; HyClone, USA) with 10% fetal bovine serum (FBS; Cat#FSP500, ExCell Bio, China), 100 U/mL penicillin, and 100 mg/mL streptomycin (Sigma Aldrich Saint Louis, MO, USA). The cells were cultivated in a humidified atmosphere at 37 °C with 5% CO_2_.

### RNA isolation, reverse transcription, and qRT-PCR

Total RNA of cells and tissue samples was extracted by TRIzol™ reagent (Ambion, Thermo, MA, USA). Concentration and purity of the RNA was examined by Nanodrop 2000c spectrophotometer (Thermo, MA, USA). Reverse transcription was followed the manuscript of miRcute Plus miRNA first-strand cDNA synthesis kit (TIANGEN BIOTECH, Beijing, China) and the ReverTra Ace qPCR RT master mix with gDNA removal kit (Toyobo, Japan). qRT-PCR analysis was carried out triplicate using either the miRcute Plus miRNA qPCR kit (SYBR Green) (TIANGEN BIOTECH, Beijing, China) or the THUNDERBIRDTM SYBR qPCR mix (Toyobo, Japan). The following primers were used: Pri-miR-486-3p F: ACCAGGCCAAGGCTTAGCTT and R: ACCCTGACGGTCTCCTGACT; hsa-miR-486-3p F: GGGGCAGCTCAGTACA and R: GGTCCAGTTTTTTTTTTTTTTTATCCT; U6 F: ATTGGAACGATACAGAGAAGATT and R: GGAACGCTTCACGAATTTG; BCL6 F: GTCCTGCAGCAGTAAGAATGCCTG and R: GGCTGTTGAGCACGATGAACTTGT; Snail F: ACCCCAATCGGAAGCCTAAC and R: TCCCAGATGAGCATTGGCA; GAPDH F: CTTTGGTATCGTGGAAGGACTC and R: GTAGAGGCAGGGATGATGTTCT.

### Irradiation and transfection

6 Gy of ^60^Co γ-rays was used to irradiate the cells. siRNAs and miR-486-3p mimic/inhibitor were designed and purchased from GenePharma (Suzhou, China). The sequences are as follows: miR-486-3p mimic sense: CGGGGCAGCUCAGUACAGGAU and antisense: CCUGUACUGAGCUGCCCCGUU; miR-486-3p Inhibitor: CGGGGCAGCUCAGUACAGGAU. siBCL6: AGTGAAGCAGAGATGGTTT; siSnail: CAGAUGUCAAGAAGUACCATT; BCL6 overexpression plasmid were synthesized by Fenghui Biologicals (accession ID: NM_001130845). miR-486-3p promoter wild-type /mutation and Snail wild-type /mutation were obtained from TSNGKE Biotech. Cat numbers are listed below: WT-miR-486-3p promoter: Y0040634-5; MUT-miR-486-3p promoter: Y0040634-6; WT-Snail: Y0040634-1; MUT-Snail: Y0040634-2. Considering a 60 mm dish as an example, 100 nM siRNA and 4 µg/mL plasmids were transfected with lipofectamine 2000 (Invitrogen, Carlsbad, CA, USA). The medium was replaced 6 h later and the cells were harvested 24 h or 48 h later.

### Mice and mice treatment

Male C57BL/6 mice (6–8 weeks) were provided by Vital River Laboratory Animal Co (Beijing, China). The mice were reared in standard animal feeding environment. 25 Gy of ^60^Co γ-rays chest irradiation was used to establish RIPF model. Animals were randomly assigned for four groups (20 mice per group) and lung tissues were collected at 1, 2, 3, 4 month after irradiation. For the special groups of mice, we used Adeno-associated virus (AAV, purchase from GENE, Shanghai, China) for nasal feeding in the dosage of 6.84 × 10^10^ vg/mouse.

### Western blot analysis and antibodies

Total proteins in cells and tissues were extracted with the commercial protein extraction reagent (Thermo, MA, USA, Cat#78501 and Cat#78510) for 30 min on ice. Then, centrifuge the contents at 4 °C, 12,000 rpm for 15 min and collect the supernatant. The reagent was supplemented with protease inhibitor cocktail (Bimake, Shanghai, China, Cat#B14001, 1:100). Bicinchoninic Acid Assay (BCA) (TIANGEN, Beijing, China, Cat#PA115) was used for measured the protein concentrations and 40 μg whole-cell lysates was used for western blot analysis. The antibodies used in this analysis were: anti-E-cadherin (CST; USA; Cat#3195S; 1:1, 000); anti-N-cadherin (CST; USA; Cat#13116S; 1:1, 000); anti-Vimentin (Abcam; USA; Cat#ab8978, 1:1, 000); anti-Snail (CST, USA, Cat#3895S, 1:1,000); anti-GAPDH (SXanta Cruz, USA; Cat#sc-25778, 1:1, 000); and anti-BCL6 (Proteintech; USA; Cat#21187-1-AP, 1:1000). The immunoreactive signals were visualized by chemiluminescence agent (Thermo, MA, USA) and the results were quantified by ImageJ software (Bethesda, MD, USA).

### Immunofluorescence analysis

Cells were fixed in 4% paraformaldehyde for 30 min and then followed by permeabilization using 0.3% TritonX-100 for 30 min at room temperature. After washing 3 times with PBS, cells were blocked in 10% FBS in PBS at room temperature for 1 h and incubated with primary antibody overnight at 4 °C. The antibodies used in this experiment were as follows: E-cadherin (CST, 3195S; 1:500); N-cadherin (CST, 13116S; 1:500). The next day, after washing 3 times with PBS, cells were incubated with fluorescence-labeled secondary antibodies (Invitrogen, Carlsbad, CA, USA, Cat#A21202/A11037). Blocker containing DAPI (ZSGB-BIO, Beijing, China, Cat#ZLI-9557) was used for blocking. Fluorescent images were acquired using X-LIGHT V3 (CRESTOPTICS, Rome, Italy) and NIKON TI2-E (Tokyo, Japan) and the relative fluorescence densities were quantified by ImageJ software.

### Dual-luciferase reporter gene assay

The JASPAR (https://jaspar.genereg.net/) and TargetScan (http://www.targetscan.org/vert_71/) databases were used to predict transcription factors that bind to the miR-486-3p promoter region and target genes of miR-486-3p, respectively. The sequence of Snail was cloned into pmirGLO Daul-Luciferase vector. Mutation plasmids were performed in the binding sites. HEK-293T cells were inoculated in 24-well dishes at 50% confluency each well before transfection. Next day, co-transfected with 400 ng wild-type/mutation plasmid and 20 pmol miR-486-3p mimic. For the miR-486-3p promoter region, we constructed wild-type and mutant plasmids containing transcription factor binding sites. The two plasmids were co-transfected with the BCL6 overexpression plasmid into cells according to the previous method. After 48 h, the dual luciferase reporter kits (Promega, San Luis Obispo, WI, USA) were used according to the manufacturer’s instructions, and the relative luciferase activity was assayed by SpectraMax i3X (molecular device, San Jose, USA).

### Histological analysis

Mouse lung tissues were immersed in 4% paraformaldehyde for 48 h at 4 °C, embedded in paraffin and cut into 5 μm thickness. Histopathological study was made using by hematoxylin and eosin (H&E; ZSGB-BIO, Beijing, China, Cat#ZLI-9610) staining and quantitated by a semi-quantitative scoring system in Szapiel [36]. Masson’s triple stain according to the Masson’s Trichrome Stain Kit manuscript (Solarbio Life Science, Beijing, China, Cat#G1340) was used to detect the collagen deposition in lungs. All the histological figures were acquired using Nikon’s Eclipse E600 microscope (Nikon, Tokyo, Japan) and all the figures quantified using ImagePro Plus software (Bethesda, MD, USA).

### Immunohistochemistry (IHC) assay

For Immunohistochemistry, paraffin-embedded lung tissue samples were dewaxed, hydrated, and washed. After neutralization of the endogenous peroxidase, the tissue was retrieved in citrate buffer (PH = 6.0) using the high-pressure antigen retrieval method. Waiting for the cooled at room temperature, the goat serum was blocked for 1 h and then incubated with primary anti-body at 4 °C for 8–10 h. The anti-body’s information as followed: anti-Snail, CST, USA, Cat#3895S, 1:200. The compatible secondary antibody was incubated for 1 h at room temperature. Sections were developed with DAB working solution (ZSGB-BIO, Beijing, China, Cat#ZLI-9019) and hematoxylin solutions were used to dye the pathological sections. All tissues were observed by microscope (Olympus, Tokyo, Japan) at 200× magnification. ImageJ was used to quantify the staining results, and H score was assigned [37].

### Statistical analysis

All data were shown as mean ± SD and were calculated from three replicate experiments. Statistical analysis between two group was performed using t-test, and among the three or more groups using one-way analysis of variance (ANOVA). The data were analyzed using the IBM SPSS Statistics for Windows, v25 (IBM Corp., Armonk, N.Y., USA). The differences were considered significant at *p *< 0.05.

## Results

### miR-486-3p is significantly downregulated after the radiation treatment

**Fig. 1:**
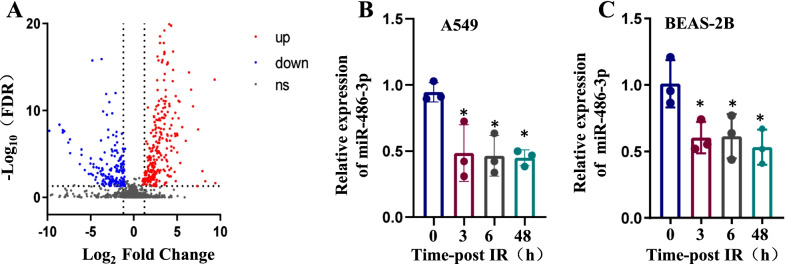
Ionizing radiation can reduce the expression of miR-486-3p. **A** A volcano plot representing differential miRNA expression between control and irradiation groups in BEAS-2B cells. **B** After irradiating A549 cells with 6 Gy ^60^Co γ-rays, the content of miR-486-3p was examined at 0 h, 3 h, 6 h and 48 h using qRT-PCR technique. **C** After 6 Gy irradiation of BEAS-2B cells, the levels of miR-486-3p were detected. *p < 0.05 compared with the control group

In a recent work conducted by our research group, it was discovered that 6 Gy irradiation might promote EMT and modify the concentration of miRNAs in both A549 and BEAS-2B cells. [13, 38]. Lung tissues were used for miRNA microarray analysis 14 days after mice received single irradiation in the whole chest to screen out miRNAs involved in this process, and miRNAs that might be involved in ionizing radiation-induced lung epithelial-mesenchymal transition were screened by miRNA sequencing analysis. miRNAs differentially expressed (more than two folds of change) in the irradiated and control groups are shown in Fig. 1A. qRT-PCR was applied to observe miR-486-3p’s levels at 0 h, 3 h, 6 h, and 48 h after the irradiation (Fig. 1B, C).

### Suppression of miR-486-3p could promote EMT occurrence

**Fig. 2:**
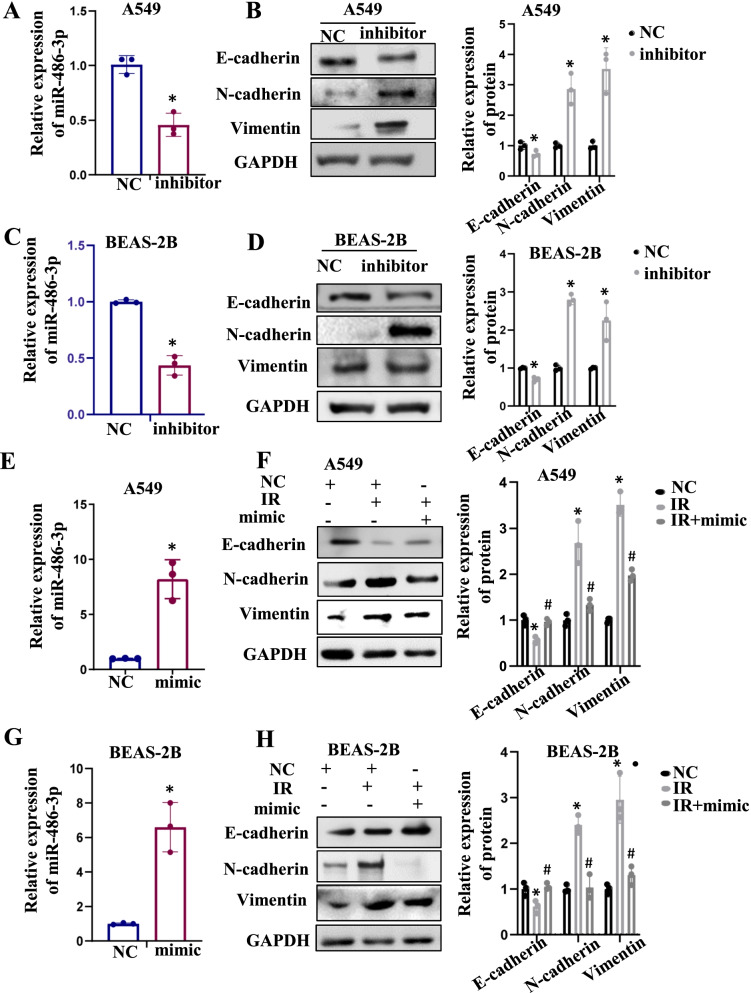
IR downregulates miR-486-3p to promote EMT. **A** qRT-PCR was performed for verifying the transfection efficiency of miR-486-3p inhibitor in A549 cells. **B** EMT-related protein was checked by Western Blot. On the right, bar graph shows the quantitative analysis of the protein using ImageJ. **C** qRT-PCR was performed for verifying the transfection efficiency of miR-486-3p inhibitor in BEAS-2B cells. **D** EMT-related protein was checked by Western Blot in BEAS-2B cells. On the right is the protein quantitative protein mapping. **E** Transfection efficiency of miR-486-3p mimic in A549 cells and BEAS-2B cells (**G**). Detection of EMT-related proteins after transfected miR-486-3p mimic in A549 (**F**) and BEAS-2B cells (**H**), and the bar graph showed gray value analysis. *p < 0.05 compared with NC, ^#^*p* < 0.05 compared with IR group.

Authors it is well recognized that IR cause the cells to undergo the EMT process. Whether this phenomenon was associated with miR-486-3p was investigated. In this investigation, an inhibitor of miR-486-3p was transfected into both cells. Transfection efficiency was detected by qRT-PCR (Fig. 2A, C, left). Western blot was executed to check the alterations in epithelial-mesenchymal marker (Fig. 2B, D, right). The results showed that epithelial markers decreased and mesenchymal markers increased when transfected with miR-486-3p inhibitor, which implying that the occurrence of EMT. As depicted in Fig. 2E–H, although there was clear evidence of the occurrence of the EMT process in the IR group compared to the control group, increase in the E-cadherin level and decreases in the N-cadherin and Vimentin levels were observed after miR-486-3p overexpression. This recommended that miR-486-3p overexpression could prevent the IR-induced EMT. The above experiments comprehensively demonstrated that miR-486-3p is crucial for the IR-induced EMT process.

### miR-486-3p suppressed Snail expression via binding to the 3′-UTR region

**Fig. 3:**
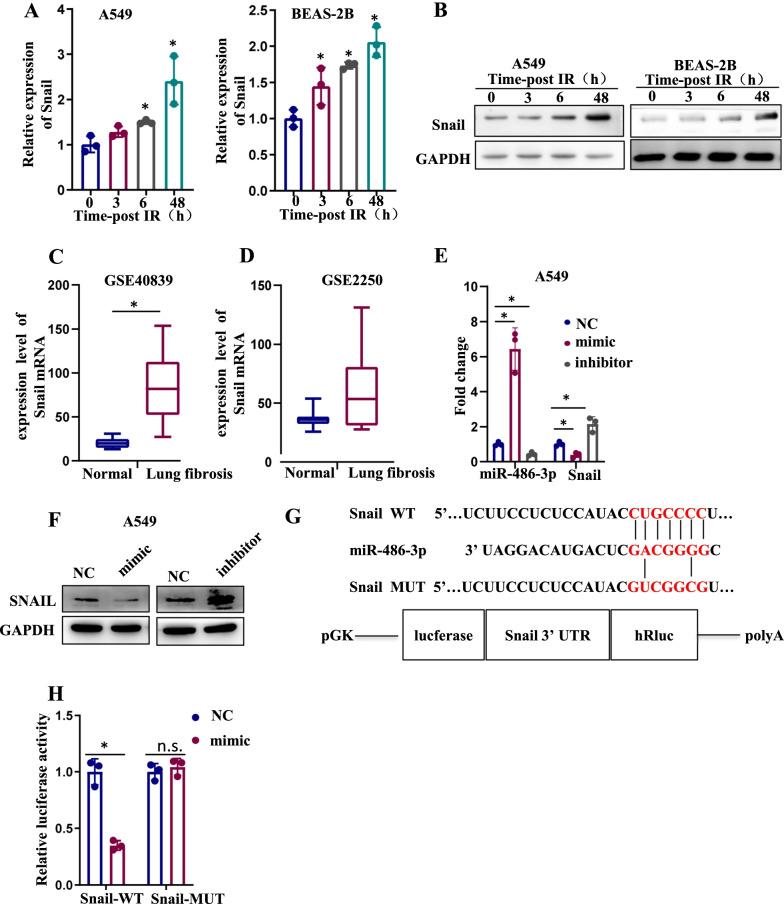
miR-486-3p directly target Snail. **A** The mRNA of Snail is detected by qRT-PCR in both cells after 6 Gy irradiation at 0 h, 3 h, 6 h, 48 h. **B** Detection of protein changes of Snail at 0 h,3 h,6 h,48 h after irradiation in both cells. **C** Mice (GSE2250) and **D** human (GSE40839) lung fibrosis models (https://www.ncbi.nlm.nih.gov/geo/query/acc.cgi) show the change of Snail proteins. **E** The change of miR-486-3p and Snail mRNA levels were checked by qRT-PCR after transfected miR-486-3p inhibitor and mimic. **F** The protein change of Snail after transfected miR-486-3p inhibitor and mimic. **G** Putative binding site of miR-486-3p within the 3′UTR region of Snail and mutant sequences. **F** Results of dual luciferase reporter activity assays in HEK-293T cells. *p < 0.05

We use the TargetScan to predict candidate target genes of miR-486-3p. We focused on Snail and checked the expression after irradiation. Both mRNA and protein levels showed that it was highly expressed after irradiation (Fig. 3A, B). Next, we examined the levels of Snail in mice (GSE2250) and human (GSE40839) lung fibrosis models and obtained consistent findings (Fig. 3C, D). When Snail expression was examined in both cells, decreased protein levels were observed in the miR-486-3p mimic group, while increased levels in miR-486-3p inhibitor group. (Fig. 3E, F). The binding sites are depicted in Fig. 3G. We co-transfected miR-486-3p mimic and Snail-WT/Mut plasmids into HEK-293T cells. As shown in Fig. 3H, miR-486-3p mimics greatly reduced the luciferase activity of Snail-WT, but this phenomenon did not occur in the Snail-Mut group.

### miR-486-3p suppressed radiation-induced EMT by targeting Snail

**Fig. 4:**
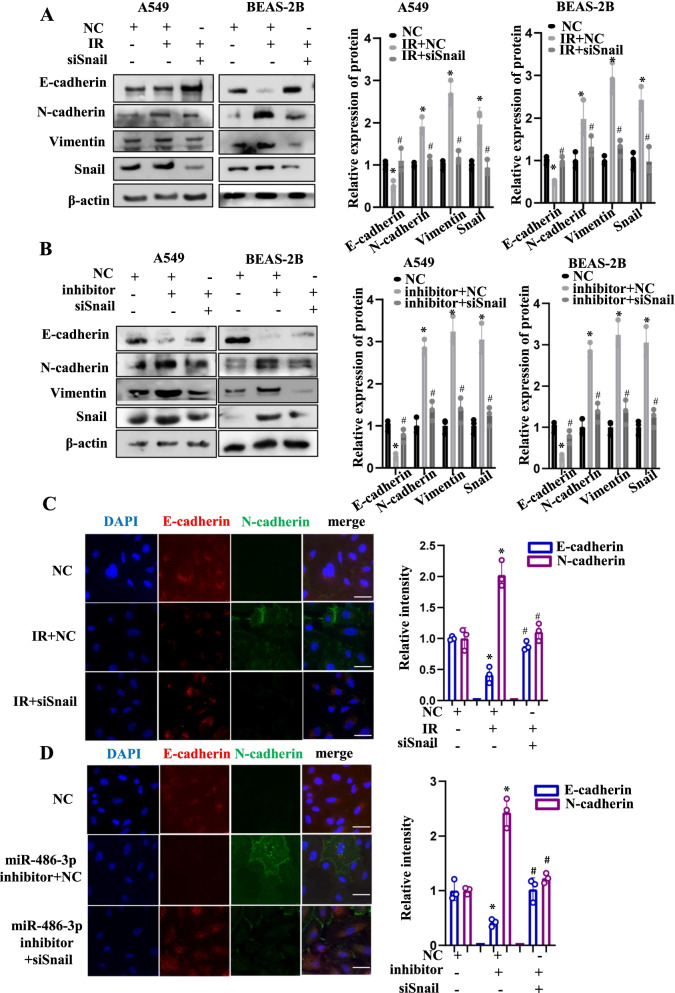
The siSnail inhibits IR-induced EMT and knockdown miR-486-3p-induced EMT. **A** Transfection of siSnail after irradiation of both cells using 6 Gy and detection of EMT-related protein expression. The bar graph shows the gray value analysis. **B** Transfection of miR-486-3p inhibitor and siSnail in both cells to examine changes of EMT-related proteins and the bargraph shows the gray value analysis. **C** In A549 cells, immunofluorescence experiments were used to analyze E-cadherin and N-cadherin changes in knockdown Snail after IR and IR. The histogram is a quantitative analysis of the relative fluorescence intensity and area. The scale bar represents 50 µm. **D** In A549, transfection with miR-486-3p and then knockdown of Snail was examined for changes in E-cadherin and N-cadherin. The scale bar represents 50 µm. On the right is the quantitative analysis of fluorescence intensity and area. *p < 0.05 compared with NC, ^#^*p* < 0.05 compared with IR + NC or inhibitor + NC

To confirm whether miR-486-3p was regulated the EMT process through Snail, siSnail was transfected post-IR or co-transfected with miR-486-3p. It was observed that Snail knockdown after irradiation significantly increased E-cadherin and caused the rebound of N-cadherin and Vimentin (Fig. 4A). As depicted in Fig. 4B, in the group with miR-486-3p and Snail co-knockdown, a rebound of the epithelial marker occurred, and the mesenchymal markers were further decreased, indicating that siSnail could significantly inhibit the EMT process induced by the knockdown of miR-486-3p. Furthermore, immunofluorescence analysis was performed. Red fluorescence indicated E-cadherin, and green fluorescence indicated N-cadherin. Upon irradiation or miR-486-3p knockdown, red fluorescence was diminished, while the area and the relative fluorescence intensity of green fluorescence were increased. However, upon Snail knockdown in both conditions, the area and the relative fluorescence intensity of red fluorescence were higher compared to those observed upon irradiation or miR-486-3p knockdown. In contrast, green fluorescence was weakened after the Snail knockdown (Fig. 4C, D, left). Besides, a quantitative analysis of relative fluorescence was performed by ImageJ software (Fig. 4C, D, right). In short, the knockdown of Snail significantly inhibited the process of EMT induced by either irradiation or miR-486-3p knockdown.

### BCL6 inhibited miR-486-3p expression at the transcriptional level

**Fig. 5:**
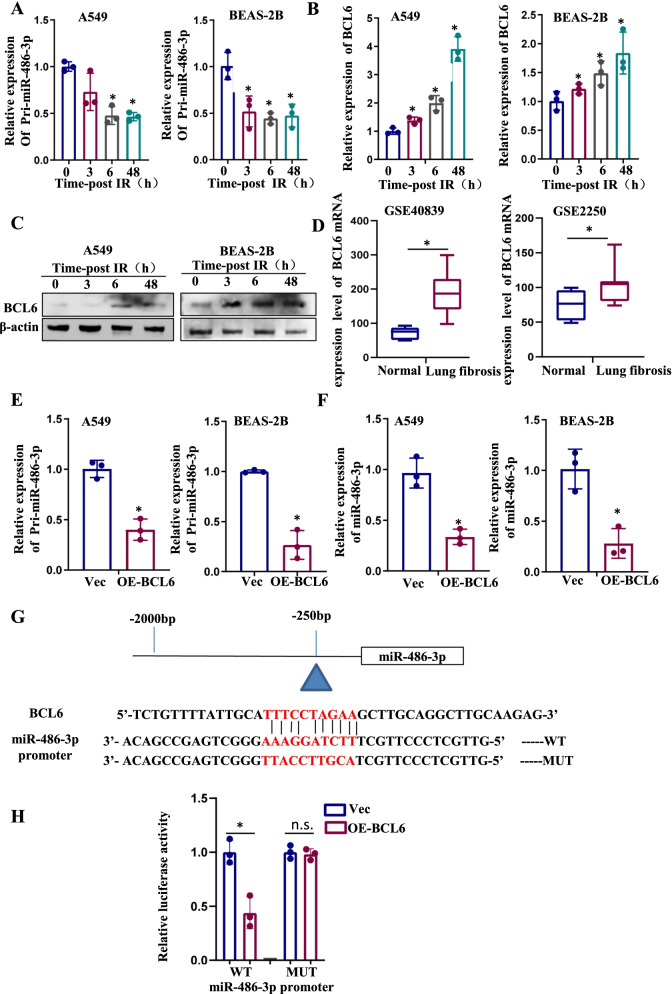
IR actives BCL6 which regulates miR-486-3p transcription. **A** Detection of pri-miR-486-3p expression at different time after irradiation of both cells using qRT-PCR. **B** Detection of BCL6 mRNA expression at different time after irradiation using qRT-PCR. **C** Changes of transcription factor BCL6 protein levels at different time after irradiation. **D**, **E** Changes of pri-miR-486-3p and miR-486-3p after overexpress BCL6 in both cells. **F** Schematic diagram of the binding site of BCL6 and miR-486-3p promoter region. **G** Dual luciferase reporter system validates the binding site. *p < 0.05

To find the reason for the decrease of miR-486-3p, we first examined the content of pri-miR-486-3p after irradiation. As shown in Fig. 5A, irradiation reduced pri-miR-486-3p levels in different cells. According to the JASPAR and animal TF databases, BCL6 binding site is present on the miR-486-3p promoter. Thereafter, the effect of IR on BCL6 was investigated, which revealed an increase in the expression of BCL6 after irradiation in both A549 and BEAS-2B cells (Fig. 5B, C). Similarly, we found increased expression of BCL6 in the datasets of mouse (GSE2250) and human (GSE40839) lung fibrosis models (Fig. 5D). BCL6 was overexpressed in both A549 and BEAS-2B cells, and subsequently, the expressions of pri-miR-486-3p and miR-486-3p were evaluated. The qRT-PCR results revealed that upon BCL6 overexpression, the production of both pri-miR-486-3p and miR-486-3p exhibited a significant decrease (Fig. 5E, F). Moreover, the binding site between miR-486-3p promoter region and BCL6 was predicted (Fig. 5G). The results of the dual luciferase reporter system showed that the relative fluorescence of the wild-type miR-486-3p promoter was significantly reduced after BCL6 overexpression, while little change occurred in the control group (Fig. 5H). Therefore, it was inferred that BCL6 exerted a repressive effect on the promoter region of miR-486-3p. BCL6 inhibits the activity of the miR-486-3p promoter region, thereby weakening the transcription process.

### BCL6 promoted EMT via the BCL6/miR-486-3p/Snail axis upon IR

**Fig. 6:**
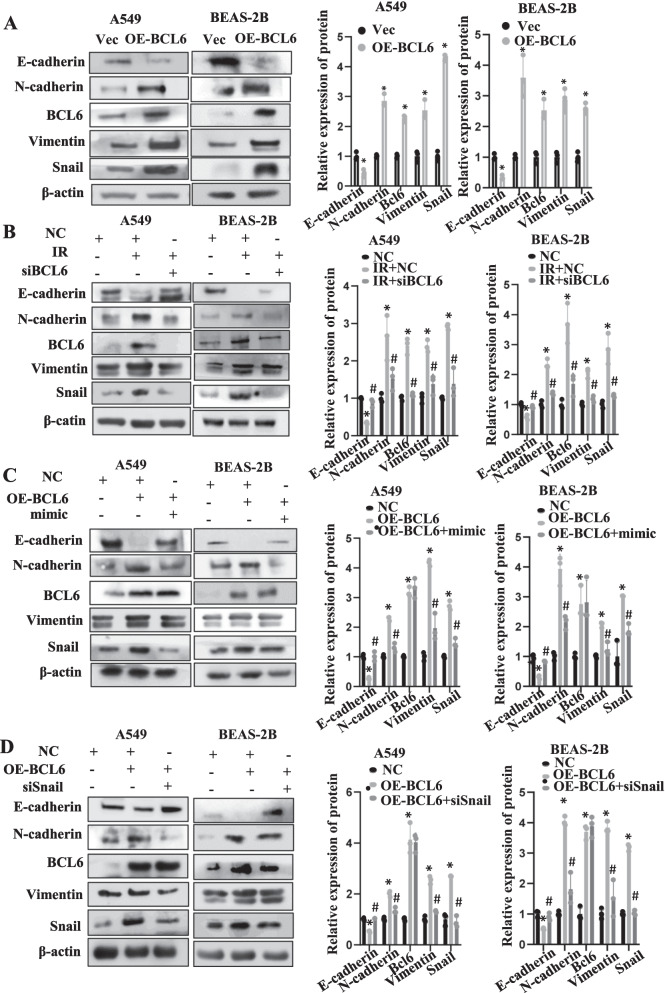
BCL6 promote EMT by repress miR-486-3p expression following IR. **A** Overexpression of BCL6 in two types of cells to check the changes of EMT-related proteins. **B** Knockdown of BCL6 after irradiation and observation of EMT and target protein expression in two cell types. **C** Firstly, BCL6 was overexpressed in cells and then transfected with miR-486-3p mimic to check the related proteins change. **D** Overexpression of BCL6 in cells and then knockdown of the target gene Snail, observation of changes in EMT-related proteins. On the right, the protein gray value analysis was showed. *p < 0.05 compared with NC; ^#^*p* < 0.05 compared with OE-BCL6

Since BCL6 could inhibit miR-486-3p after irradiation which could promote EMT, we overexpressed BCL6 in both cells. It was found that E-cadherin was meaningfully diminished and N-cadherin, Vimentin were significantly increased, implying that the EMT process was stimulated upon BCL6 overexpression (Fig. 6A). Next, the effect of BCL6 knockdown was analyzed. It may be observed in Fig. 6b, E-cadherin, the levels of which were reduced by IR, exhibited a rebound after BCL6 knockdown. Moreover, the levels of N-cadherin and Vimentin, which increased after IR, exhibited a certain decrease upon BCL6 knockdown (Fig. 6B). Moreover, BCL6 overexpression in the cells immediately followed by overexpressing miR-486-3p. The experimental results showed that when both BCL6 and miR-486-3p were overexpressed in the cells, EMT caused by BCL6 overexpression was significantly inhibited (Fig. 6C). Subsequently, BCL6 was overexpressed in the cells, followed by Snail knockdown. The results revealed that knockdown of Snail could effectively inhibit BCL6-induced EMT (Fig. 6D). All above demonstrated that IR could reduce the expression of miR-486-3p by activating BCL6, increase the Snail levels, and promote EMT.

### Irradiation induces RIPF through the BCL6/miR-486-3p/Snail signaling axis

**Fig. 7:**
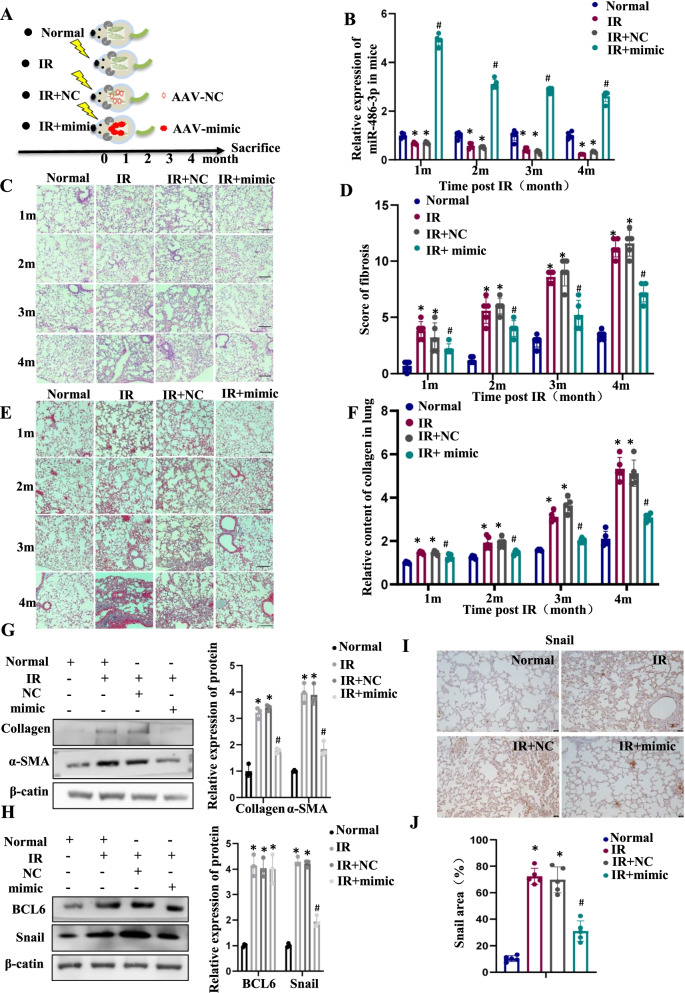
BCL 6 mediates RIPF via miR-486-3p/Snail Axis. **A** Mice were divided into four groups: Normal (Nor), irradiated alone (IR), irradiated after lung administration (AAV + NC, IR + NC), irradiated after lung administration (AAV + mimic, IR + mimic). **B** miR-486-3p levels in irradiated mouse lung tissue (n = 5) at different time using qRT-PCR. **C**, **D** H&E staining (n = 5) and the score of lung tissue, scale bar, 100 µm. **E** Masson’s staining (n = 5) and the Relative content quantitation of Collagen. **G** Alterations of pulmonary fibrosis-related protein levels in lung tissue after 4 months of irradiation. On the right, the bar graph shows the gray value analysis. **H** Changes in transcription factor BCL6 and target proteins in different groups after irradiation. **I** Snail expression in lung tissues by immunohistochemical experiment. **J** Quantitative analysis of IHC (n=5). *p < 0.05 compared with Normal; ^#^*p* < 0.05 compared with IR + NC

Irradiation (25 Gy) of mouse lungs was used to establish RIPF model and to verify whether miR-486-3p could inhibit the RIPF process in vivo. An AAV vector was used to stably carry miR-486-3p mimic specifically into mouse lung. The experimental groups of mice are shown in Fig. 7A. As shown in Fig. 7B, the miR-486-3p contents in mouse lung were examined. Compared with Normal group, the miR-486-3p levels were significantly lower in IR and IR + NC group but highly expressed in IR + mimic group. As a good indication, miR-486-3p content was continuously highly expressed after lung injection using AVV. Next, the mice lung tissue was evaluated using HE staining and marking the score (Fig. 7C, D). Through pathological changes and scores, compared with the control group, the alveolar structure of mice in the irradiation group was changed and fibroblasts were proliferated. Alveolar septum widened significantly, showing patchy fibrosis changes, pulmonary fibrosis significantly. In the Masson staining, it was observed that collagen deposition significantly increased in the irradiated group (Fig. 7E, F). However, in the IR + mimic group, alveolar density and alveolar septum thickness were meaningfully reduced compared to the irradiated group.

Next, collagen and α-SMA was examined in tissues (Fig. 7G). The results showed that the contents of the two proteins in the irradiated group were significantly higher than the unirradiated group, which indicated that the mouse RIPF model was established successfully after irradiation. However, when we overexpressed miR-486-3p in vivo, the contents of both collagen and α-SMA significantly reduced, which also proved the reversal effect of miR-486-3p on RIPF process. Furthermore, both BCL6 and Snail increased in irradiated group, while only Snail exhibited a significant decrease in IR + mimic group (Fig. 7H). In addition, Snail expression in lung tissue was detect by immunohistochemical. We found that the positive expression rate of Snail was lower than that in IR and IR + NC, which indicated that Snail was the downstream target protein of miR-486-3p (Fig. 7I, J). These results demonstrated that irradiation causes an increase in BCL6 and a decrease in miR-486-3p in lung tissue, triggering the RIPF process. When we overexpressed miR-486-3p, the content of the target gene, Snail, significantly reduced, and the degree of RIPF was also significantly reduced. This suggested that miR-486-3p could be a potential target to interfere with the RIPF process.

## Discussion

The mechanism of RIPF, one of the serious complications affecting the survival and prognosis of tumor patients, is still unknown. In the biological process, EMT converts epithelial cells to mesenchymal cells, increases fibroblast content, and accelerates RIPF [39]. Snail plays a key role in the development of RIPF as an activator of EMT [40–42]. However, the molecular mechanism of IR regulating Snail needs further investigation.

MiRNAs have been reported to play critical roles in radiation-induced lung injury [43–45]. The results of this study revealed that miR-486-3p exerts its biological effects by affecting EMT, which is a key link in the RIPF process. Several studies have suggested that miR-486-3p has several important biological functions. It has been reported that miR-486-3p is under-expressed in lung cancer. miR-486-3p mimic can reduce the weight of transplanted tumor in vivo [46]. Downregulation of miR-486-3p mediates CEMIP promoting NPC proliferation and extracellular matrix production in intervertebral disc degeneration [47]. In addition, overexpression of miR-486-3p promoted cell growth and proliferation, while knockdown of the target gene DDR1 significantly inhibited cell growth and increased apoptosis [48]. Here, miR-486-3p was identified to be a repressor of RIPF, which can decelerate the onset and development of RIPF by silencing the expression of Snail and thereby suppressing the IR-induced EMT process. The present study provides a suitable model for studying IR-induced EMT and enriches the overall understanding of the RIPF. In vivo studies showed that miR-486-3p was successfully expressed in the lung of mice by AAV vector, which could eliminate the alveolar septum thickening, tissue denseness and collagen deposition induced by irradiation. It reveals the key role of miR-486-3p in the process of RIPF and may become a potential therapeutic target for idiopathic pulmonary fibrosis.

Transcription factors are a group of protein molecules that bind to specific sequences of genes and regulate gene production at the transcriptional level [49]. In this study, we analyzed the characteristics of the promoter region of miR-486-3p and predicted the transcription factor binding domain of the promoter region using an online database. BCL6, a class of transcriptional repressors, was previously reported to promote the growth, migration, and metastatic ability of breast cancer cells [50–52]. BCL6 was first reported to be involved in the inflammatory response and can participate in the inflammatory response in vivo in conjunction with multiple inflammatory factors [53–56]. Therefore, it was highly expressed in various tumors, thereby promoting tumor invasion and metastasis [57–60]. However, its relationship with irradiation has not been reported. It is observed that irradiation-induced increase in BCL6 is one of the important reasons for the decrease in miR-486-3p caused by IR. In addition, a lot of work was carried out to confirm the regulation of miR-486-3p and its target gene Snail by BCL6. We reported for the first time that BCL6 was up-regulated in lung tissue after irradiation and affected the pathological changes of tissues by regulating the generation of miRNA in vivo. However, the regulatory mechanism of BCL6 involved in radiation-induced diseases needs more research.

## Conclusions

**Fig. 8:**
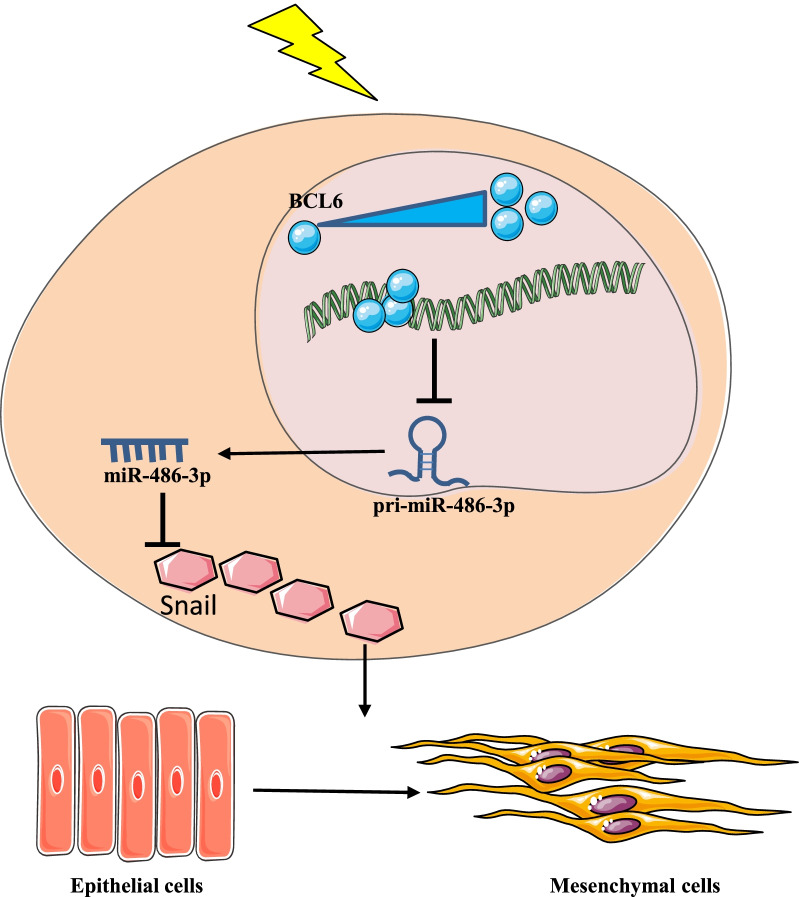
Working model of EMT induction by IR-activated transcription factor BCL6 via miR-486-3p/Snail.BCL6 was activated and increased after IR, and binding to the promoter region of miR-486-3p inhibited the transcription of miR-486-3p, resulting in a decrease in pri-miR-486-3p content. pri-miR-486-3p reduction led to a decrease in miR-486-3p content and weakened the silencing effect of miR-486-3p on Snail. Thus, the above figure describes a complete Radiation-induced pulmonary fibrosis through the BCL6/miR-486-3p/Snail axis

As indicated by Fig. 8, our study provides definitive evidence that IR caused a decrease in miR-486-3p, and suggests that this decrease was due to an increase in transcription inhibitory factor BCL6. Our study also demonstrated that miR-486-3p inhibits the EMT process by inhibiting its target gene Snail; thus, alleviating the RIPF. Our result is very consistent because both in vivo and in vitro reached the same conclusion. Although this study demonstrated that changes in the BCL6/MIR-486-3p/Snail axis induced EMT and promoted the formation of RIPF, it is still far away from elucidating the mechanism of RIPF. We believe that in the future, there will be more experimental studies to fully explain the development of the RIPF that could help patients suffering from the disease.

## Data Availability

The data is available on the request.

## Abbreviations

RIPF: Radiation-induced pulmonary fibrosis
EMT: Epithelial–mesenchymal transition
IR: Ionizing radiation
miRNA: MicroRNA
3′-UTR: 3′-Untranslated region
BCL6: B cell lymphoma 6 protein
α-SMA: Alpha-smooth muscle actin
A549: Human alveolar type II epithelial cancer cell
BEAS-2B: Human normal lung epithelial cell
HEK-293T: Human embryonic kidney cells
qRT-PCR: Real time quantitative polymerase chain reaction
siRNA: Small interfering RNA
AAV: Adeno-associated virus
NC: Negative control
ANOVA: One-way analysis of variance
